# The Institutional Worker

**Published:** 1913-03-01

**Authors:** 


THE
### INSTITUTIONAL WORKER
Being a Special Supplement to "The Hospital"

### Pensions for Asylum Workers.

BY the Act of 1909 a pension can be secured at the age of 55 years, if 20 years' service has been given. This presses very hard on many women who have worked in asylums from the age of 20 or under, because it demands 35 years of work before the pension age is reached. Few women can stand the strain of asylum work for this length of time. The proposal to which you allude was contained in the Asylum Officers' Bill, and aims at securing a pension for female asylum workers after 25 years of service, irrespective of age.—R. S. T.

### Holiday Home for the Blind.

WRITE to the Secretary, Mayfield House, St. Peter's Road, St. Leonards-on-Sea. The charge is 10s. a week for adults, or free with subscriber's letter. As a rule the blind are happier when they mix with ordinary convalescents, but as this particular case is helpless and nervous a special Home will be more suitable.—VERA.

### Nursing Institute at Montreux.

THERE is no very large demand for nurses at Montreux. When required they command about £4 a week. Write to The English Nurses' Institute, c/o Mrs. Bell, Clarens, Montreux, enclosing postal coupon for reply. Of course you understand that Montreux is the general name applied to the villages along the east shore of the Lake of Geneva, from Clarens to Veytaux. It is reckoned that from 30,000 to 40,000 foreigners visit the district, of whom over a third are English-speaking.—BETA.

### Convalescent Patient's Diet.

A CONVALESCENT patient would receive tea or cocoa with bread and butter for breakfast and tea, milk or soup for lunch; meat, potatoes, bread and pudding for dinner; soup and bread for supper. To this, however, might be added bacon for breakfast, greens for dinner, an egg for tea, an extra pint of milk. The cost of the ordinary diet would be at present prices 4s. 8d. Extras, exclusive of greens, which can be obtained from the garden, would cost an additional

### OUR BUREAU OF INFORMATION.

### Rules for Correspondents.

1. Every letter, on whatever subject, must be accompanied by the coupon to be cut from the back cover (inside page) of THE HOSPITAL, current issue, and must contain the name and address of the correspondent with pseudonym for publication if desired.
2. Replies by post cannot be given, save under exceptional circumstances at the Editor's discretion.
3. Letters from Approved Homes in reply to special needs published in the Bureau should state terms and full particulars, and be sent prepaid under cover to the Editor of the Bureau with name written across coupon for identification.
4. Proprietors of Homes which have not yet been entered on the List of Approved Homes, but have spare accommodation likely to suit special needs, are invited to write for an application form for registration. The fee for registration, which includes two announcements of the Home in the Bureau and other privileges, is 5s.
5. All communications to be addressed to the Editor of THE HOSPITAL, 28 Southampton Street, Strand, London, W.C., and marked "Bureau of Information."

### Invitation to Readers.

**The Editor is prepared to forward to applicants, without fee on either side, offers of accommodation from Homes on the Approved List suitable for medical and surgical cases, for the aged, for chronic or mental patients, for electrical treatment and massage, for convalescents and for children, at varying terms, in any required locality. Reduced terms can often be arranged. The special needs of any who may be sick or in difficulties are also made known in these columns free of charge.**

### Approved Homes Recently Added to List.

NOTE.—*No Home is added to the List without medical and other testimony to its good management.*

**Broadstairs.**—Two patients only received by experienced trained nurse, ladies or children. Special advantages for chronic invalids, deaf or blind patients. Pleasant sunny situation. Moderate terms.—DOMEN.

### Approved Homes Discontinued.

Miss Richardson, Landholm, Totland Bay; Miss Mills, Mascot, Seaton, Devon.

### Maternity Home Wanted.

WE are asked to recommend a Home in Harrogate in which a maternity patient could be received, and in which she could be attended by a Catholic doctor. Reply, stating terms and full particulars, to Mrs. R. (154A).

### Unsatisfactory Nursing Homes.

THERE is ample evidence to show, as you say, the need for giving the public some means of choosing well-managed establishments when they need nursing. It was, to use your own words, "the disgraceful and unskilful treatment some unfortunate patients have to undergo in some of the so-called Nursing Homes" which led us to formulate a system through which the public could have free access to reputable Homes without heavy fees being demanded for the introduction from the principals.—H. COOPER.

2s. 4d. This refers to a country hospital with 120 beds. The quality of food supplied is extremely good.—ELIZABETH.

### The Lenten Dietary.

As many of the workers in your institution wish to observe certain forms of self-denial during Lent consideration in matters of diet is very right. It is certain that nurses are unable to abstain from substantial food without suffering in some way, and a wise matron will make variations in the Lenten dietary whereby she may ensure that a sufficient amount of food will be taken by those who have scruples in the matter. In most institutions it is more or less a custom to provide fish for dinner on Fridays. During Lent fish could appear more often, and especially on the Wednesdays in Passion and Holy Weeks. Eggs, porridge, or sardines usually appear for breakfast on some mornings of the week, alternating with bacon, sausages, etc. By a little forethought the right dish can be offered to meet the needs of those who desire it without those uninterested in the matter being aware of any difference. Supper dishes that do not include meat can be supplied with cold meat as an alternative, and cold meat should also be available when fish is to make the staple portion of the dinner. Many who would go almost supperless on Friday, for instance, if only cold meat and pickles were provided, would make a satisfactory meal of milk pudding and bread and cheese, or of potato soup and macaroni cheese. No extra expense need be incurred. It will be found that a dietary that will meet the needs of all at this special season can be provided without increasing the bills or giving extra work in the kitchen. The expenditure of a little more forethought may be necessary, but this amount of interest will be appreciated by those who feel the need of it, especially if no comment be made.—ANGLICAN.

### A Successful Hospital Garden.

THE number of beds in this hospital is 114. The vegetables used here in December 1912 were for the staff as well as patients. We used 41½ bushels of potatoes. They were very good ones, but I do not know the market price as they were all grown in the hospital garden. Other vegetables used were: Cabbage, 10 doz. heads; savoy cabbage, 2 doz. heads; cauliflower, 1½ doz. heads; leeks, 5 doz. heads; salsify, 4 doz. heads; celery, 15½ doz. heads; Brussels sprouts, 3 bushels; kale, 2 bushels; artichokes, 8 gallons; spinach, 3 gallons; turnips, 22 gallons; carrots, 40 gallons; parsnips, 38 gallons; onions, 20 gallons, and 60 very large ones which were roasted; five or six large vegetable marrows which had been stored; beetroot, parsley, herbs, horseradish unlimited, winter cress and lettuce to make salad for 60 on one occasion. The whole of this was grown in the hospital garden. We bought 5 lb. of tomatoes, 2s. worth of watercress, one bushel of Brussels sprouts, and a sack of carrots and turnips were also given as a present.—ETHEL.

### The Cost of Vegetables.

IN endeavouring to answer your question No. 1 (for February) I have below given not only the price of the potatoes, but the prices paid for all the other vegetables, together with the quantities consumed. The stores for the nursing staff are kept quite apart from the hospital and the figures relating thereto are therefore shown separately.

*Vegetables—December* 1912.

*Nurses' Stores.*

| | | | £ | s. | d. |
|---|---|---|---|---|---|
| Potatoes* | ... | 5 loads ... | 2 | 15 | 0 |
| Savoys ... | ... | 1 doz. ... | 0 | 1 | 6 |
| Cauliflowers | ... | — | — | | |
| Carrots ... | ... | 5 stones ... | 0 | 3 | 4 |
| Turnips ... | ... | 5 stones ... | 0 | 2 | 1 |
| Tomatoes | ... | 21½ lb. ... | 0 | 10 | 9 |
| Onions ... | ... | 4 stones ... | 0 | 5 | 0 |
| Celery ... | ... | 51 roots ... | 0 | 5 | 11 |
| Sprouts ... | ... | 43 lb. ... | 0 | 6 | 0 |
| Beetroot... | ... | 1 stone ... | 0 | 1 | 0 |

*Hospital Stores.*

| | | | £ | s. | d. |
|---|---|---|---|---|---|
| Potatoes* | ... | 23½ loads ... | 12 | 18 | 6 |
| Savoys | ... | 30 doz. ... | 2 | 2 | 10 |
| Cauliflowers | | 70 ... ... | 0 | 17 | 0 |
| Carrots | ... | 15 stones ... | 0 | 10 | 0 |
| Turnips | ... | 21 stones ... | 0 | 8 | 9 |
| Tomatoes | ... | 16 lb. ... | 0 | 8 | 0 |
| Onions | ... | 3½ stones ... | 0 | 4 | 4 |
| Celery | ... | 65 roots ... | 0 | 7 | 1½ |
| Sprouts | ... | 1 stone ... | 0 | 1 | 3 |
| Beetroot | ... | 1 stone ... | 0 | 1 | 0 |

* At 11s. per load of 18 stones.

Included in the hospital stores are all the vegetables used for the medical and domestic staff. All vegetables are purchased none being grown in the hospital grounds. The average number of beds occupied for the month of December was 301. The nursing staff numbers 100, the domestic staff 50, and the resident medical men total nine. —A NORTHERN SECRETARY.

### Apartments for Elderly Ladies.

THE applicants are sisters aged respectively seventy-three and seventy, and one has been mentally afflicted but is now well. They have very small means, and desire to be accommodated at a nurse's house, where they could have two unfurnished rooms with attendance for 10s. a week, and get a little attention if ill. The district of Putney preferred. Possibly they might get taken in at the Alexandra Home. We recommend you to go and see Mrs. Perrin, 153 Merton Road, Wimbledon. She has recently moved into a larger house, and may have spare accommodation.—CONSTANCY (153x).

### Generous Help for Bureau Case.

OUR hearty thanks are due for the postal order you send us for the help of the young woman (151x) suffering from spinal and hip disease, for whom we appealed in last week's Bureau. There is little doubt that the sum necessary to ensure her being received in a Home could be secured if a vigorous effort were made locally on her behalf, and your very friendly help will stimulate those interested to make the attempt. —C. T. L.

### Mental before General Training.

IN one sense mental training may be considered a drawback as a preliminary from the fact that three years of asylum life usually leaves the nurse disinclined to begin all over again as a novice and take general training. Write yourself to one of the well-known Mental Hospitals and ask them to send you a form of application for training, with the conditions of service.— M. G. B., Herne Hill.

### Institution Facts and Figures.

### Questions for March.

1. GIVE the estimated cost per head per week for laundry expenses in your institution (*a*) for patients, (*b*) for staff. Mention the number of beds, whether acute medical and surgical cases are received, and whether the washing is done on the premises or by contract outside.

2. Taking any week in the winter months, state what weight of meat was consumed in the institution, less bacon and ham. Divide this amount by the total number of inmates on meat diet, and thus show the average weight of butcher's meat consumed per head per week. In estimating the numbers include both staff and patients, deducting only those on milk or fish, whose diet does not include beef-tea.

The figures must be the result of actual observation and computation, not estimates. Either or both of the questions may be answered by each contributor. The best answers will be published, and for each contribution considered by the Editor to be of sufficient interest for publication payment of FIVE SHILLINGS will be made.

RULES.

The following rules must be observed by all those answering the Housekeeping Questions :—

1. Answers must be written on one side of the paper. They must bear the name and address of the sender and be accompanied by coupon to be cut from the back cover (inside page) of the current issue of THE HOSPITAL. A pseudonym must be chosen if the name is not to be published.

2. Answers must be addressed to the Editor of THE HOSPITAL, 28-29 Southampton Street, Strand, London, W.C.; they must be marked in the left-hand corner "Facts and Figures," and must be received before the end of the month.

### The Cost of the Convalescent.

OUR middle diet here, which may be taken as one for convalescent patients, is as follows: Bread, 12 oz., ¾d.; butter, 1 oz., ⅞d.; meat (beef, mutton, or mince), 4 oz., 2d.; fish, 8 oz., plaice 4d., cod 3d.; potatoes, 8 oz., ¾d.; milk, 1 pint, 1⅛d.; mutton broth, 1 pint, 1½d.; beef-tea, 4½d.; rice pudding, ¾d.; tea, ¼ oz., with 2½ oz. of milk and ¾ oz. sugar, 1¼d. Extras which are prescribed by the medical staff may be added as follows: Chop, 4½d.; rabbit, 3¾d.; greens, ½d.; custard, 1½d.; extra milk, 1⅛d.; egg (present), 1¾d.; chicken, 8¼d.; plaice, 4d.; Yorkshire pudding, ¼d.; extra beef-tea, 4½d.; cream, 1d.; bacon, 1¾d. In order to explain the method of charging our wards with the actual cost per day, I have attached a copy of our actual diet sheet for January 30 last. It may be noticed that there are no entries for tea, coffee, and sugar. These are issued once weekly, and the cost is charged to each ward on the day they are dealt out. It may be found that the prices paid for vegetables are somewhat higher than in some hospitals; nothing but the best quality of goods, however, are purchased. It would be of considerable advantage to know the methods of other institutions, and I am sure the Editor would welcome a discussion on this important matter.—A NORTHERN SECRETARY.

### The Editor's Letter-Box.

### Communications have been received and attended to from:—

Mrs. S., Musselburgh; Mrs. W., Worcester; Miss F. M. E. B., Norwich; Mrs. C. R., Bexhill-on-Sea; Miss E. M. W., Broadstairs; Miss W., Highgate Park; Miss R. C., Chelsea; Miss F. M. S., Cardiff; Miss A. S., St. Leonards; Mrs. Stubbs; T. H. Cooper, Esq.; N. F. Ticehurst, Esq., F.R.C.S.; Dr. Mackussack.

The coincidence is certainly remarkable, and is annoying also, for we have taken some trouble to place all three patients. We note your request for relating to printed card of Approved Homes, and have it under consideration.—O. S.

Glad you have found two suitable Homes to choose from when the time comes, in response to your request for accommodation.—A. E. N.

### Queen Charlotte's Hospital in Difficulties.

The annual meeting of governors and subscribers of Queen Charlotte's Hospital was held on the 25th ult. The report showed that during the past year 1,880 patients had been admitted, being the largest number ever received in one year. In addition 2,367 patients had been attended and nursed in their own homes. The income was insufficient to meet the expenditure to the extent of £1,257, and as there was also a deficiency of £1,970 in 1911, the present debt was £3,227. A preliminary training school had been opened in connection with the Midwifery Training School, which it was expected would prove to be a very important feature in the training of midwives and nurses. In moving the adoption of the report, the Chairman appealed for increased support to meet the outstanding liabilities and to provide a much-needed new out-patient department, towards which a donation of £200 had recently been made. The annual meeting of the Ladies' Association of the hospital was held subsequently, and it was stated that considerable progress had been made by the Association, of which there were now over 200 members.

### A Hospital Insufficiently Known.

The Earl of Erroll, presiding at the annual meeting of the East London Hospital for Children, said he wished to appeal very strongly through the Press to the general public for more liberal support for that hospital. What seemed to him to be the crux of the whole matter was the position of the hospital. From the point of view of usefulness it could not possibly be better situated. It was in the very poorest part of London, and among a population that could not afford to pay for medical or surgical treatment. But, on the other hand, from a financial point of view, the hospital suffered considerably from its position. The other big children's hospitals were known to everybody in the West End; they were under their eyes, and they knew the work they were doing. If, however, one mentioned the East London Hospital for Children to people in the West End—and it was from the West End that they must get all their money, for there was none in that part of the town—they would say, "We never heard of it." If Shadwell were mentioned, they would also say, "We never heard of it." It would be a great thing if they could get people to come down and see how the hospital was working.

### Manager's Notices.

**Letters relating to the publication, sale, and advertising departments of "The Hospital" must be addressed to The Manager, "The Hospital," The Hospital Building, 28 and 29 Southampton Street, London, W.C.**

Subscriptions may begin at any time, and are payable in advance. Cheques and Post-Office Orders should be crossed London County and Westminster Bank, Covent Garden Branch, and made payable to the Manager of The Hospital.

Rates of Subscription (including Postage).

| *United Kingdom.* | | *Foreign and Colonial.* | |
|---|---|---|---|
| Three Months ... ... | 2s. 0d | Three Months ... ... | 2s. 6d |
| Six Months ... ... | 4s 0d | Six Months ... ... | 5s. 0d |
| Twelve Months ... ... | 6s 6d | Twelve Months ... ... | 8s. 8d |

### Institutional Needs.

### OPERATING THEATRE EQUIPMENT.

(M. Schaerer A.G., 41 Berners Street, W.)

This well-known Continental (Swiss) firm of hospital equipment manufacturers have submitted to us a catalogue of their technical novelties and surgical instruments in general. Without any derogation to British instrument-makers, the best of whom are thoroughly efficient and reliable, it is only simple justice to admit that the extraordinary ingenuity of many of the Schaerer A.G. products exceeds anything that can be found indigenous in this country. Some of these instruments and theatre accessories are of the nature of freak appliances, designed by some particular surgeon to suit his own methods of operating; but this is true only of a small proportion, and the majority of the instruments figured are absolute necessities in certain special branches of surgery, such as that of the lungs, the blood-vessels, the nerves, and so on. One particularly ingenious instrument is Florian Hahn's suture apparatus for gastric and intestinal resections. An intestinal clamp forms part of this machine, and is placed in position close to the intended line of resection. The gut is then cut close to the clamp, and a lever is pressed, which places in gear the suture apparatus, threaded beforehand. The wall of the viscus is then mechanically sewn up in a fraction of the time taken to do it by hand. It is stated that this machine is simple in construction, safe in execution, and easily learned. It is priced at £8 5s. This particular novelty we have not had an opportunity of testing or seeing tested in actual working. But of the wonderful Schaerer A.G. operating table we can report most favourably after an extended trial for all kinds of operations. Complicated as it looks, with a formidable array of levers, gears, and pinions, this table is very easily worked, and with a little practice can be thoroughly mastered. It provides for every conceivable kind of movement and position of the patient, and alterations can be secured during an operation without any necessity for disturbing the patient or the field of operation. Unusual features are the arrangements for lateral tilt of the table either way, for the flexion and extension of the spine (*e.g.*, during gall-bladder operations), and for the prone posture during brain or spinal-cord operations. The head-piece provided for use in the latter class of work is a hollow cup, open below, so that the head may rest face downwards and at the same time respiration and anæsthetisation are quite unhindered. There are many other special points about this table, which render it adaptable for goitre, kidney, pelvic, and other operations; but mere description of them would fail to convey an adequate idea of the lengths to which the study of surgical convenience and comfort has been carried. Any hospital secretary or committee or staff which is contemplating the purchase of a new operating table should certainly make an effort to see a Schaerer A.G. table in working order before deciding on any other pattern. This can be done at more than one London hospital to our knowledge, and notably at the Bristol Royal Infirmary amongst the provincial institutions. The same applies to the sterilising plants, autoclaves, and steam disinfectors which the same firm manufacture; and, indeed, to the whole catalogue of unique hospital, theatre and laboratory equipment. Representatives of the firm will visit intending purchasers, and estimates are prepared free of charge.

